# Efectividad de la curcumina como terapia fotodinámica para los procedimientos de endodoncia: una revisión narrativa

**DOI:** 10.21142/2523-2754-1202-2024-200

**Published:** 2024-06-27

**Authors:** Alisson Melissa Salazar Villavicencio, Mauricio Zapata-Sifuentes

**Affiliations:** 1 Universidad Científica del Sur. Lima, Perú. melissasalazars118@gmail.com Universidad Científica del Sur Universidad Científica del Sur Lima Peru melissasalazars118@gmail.com; 2 División de Anatomía y Biología Celular del Tejido Duro, Departamento de Regeneración y Reconstrucción de Tejidos, Escuela de Posgrado de Ciencias Médicas y Odontológicas de la Universidad de Niigata. Niigata, Japón. mzapata@dent.niigata-u.ac.jp University of Niigata Prefecture División de Anatomía y Biología Celular del Tejido Duro, Departamento de Regeneración y Reconstrucción de Tejidos Escuela de Posgrado de Ciencias Médicas y Odontológicas Universidad de Niigata Niigata Japan mzapata@dent.niigata-u.ac.jp

**Keywords:** antibacterianos, curcumina, endodoncia, fotoquimioterapia, antibacterials, curcumin, endodontics, photochemotherapy

## Abstract

**Introducción:**

La terapia endodóntica se realiza mediante la preparación biomecánica y la medicación intracanal; sin embargo, las bacterias residuales tienen la capacidad de adherirse a las paredes del conducto radicular. Por ese motivo, la terapia fotodinámica ha ganado popularidad gracias a su capacidad para prevenir y erradicar infecciones microbianas mediante el uso de un colorante activado por luz. Objetivo: Analizar y actualizar la información sobre el efecto de la curcumina en la terapia fotodinámica para el tratamiento de conductos.

**Materiales y métodos:**

Se realizó una búsqueda de la literatura en las bases de datos en PubMed/Medline, Scopus, EBSCO, ScienceDirect y LILACS utilizando las palabras claves “curcumina”, “cúrcuma”, “fotodinámica”, “fotoquimioterapia”, “fotorradiación”, “desinfección fotoactivada”, “desinfección de conductos radiculares”, “terapia del conducto radicular” y “endodoncia”. Los artículos estuvieron en idioma español e inglés, entre los años 2018 a 2023.

**Resultados:**

Se recopiló información de los últimos cinco años con el objetivo de actualizar el tema de estudio. Se examinaron 749 artículos utilizando criterios de inclusión y exclusión, de los cuales solo 50 cumplieron estos criterios y fueron analizados. Los estudios actuales muestran los efectos de la terapia en la contaminación del biofilm del conducto radicular con *E*. *faecalis*, y evidencia que la curcumina fotoactivada promueve la ruptura del biofilm y reducción de unidades formadoras de colonias.

**Conclusión:**

La curcumina, como fotosensibilizador, demuestra un efecto antibacteriano potencial que disminuye significativamente la viabilidad de las células microbianas y la vitalidad de las biopelículas.

## Abstract (en)

**Introduction:**

Endodontic therapy is performed by biomechanical preparation and intracanal medication; however, residual bacteria can be compromised due to their ability to adhere to the root canal walls. Therefore, photodynamic therapy has gained popularity because of its good ability to prevent and eradicate microbial infections by using a light-activated dye. Objective: Analyze and to update the information on the effect of curcumin in photodynamic therapy in root canal treatment.

**Material and Methods:**

A literature search was carried out in PubMed/MEDLINE, Scopus, Ebsco, Science Direct, and LILACS databases using the keywords "curcumin", "turmeric", "photodynamic", "photochemotherapy", "photoradiation", "photoactivated disinfection", "root canal disinfection", "root canal therapy", "endodontics" in both Spanish and English, from 2018 to 2023.

**Results:**

Information from the last five years was collected with the aim of updating the study topic. 749 articles were examined using inclusion and exclusion criteria, of which only 50 met these criteria and were analyzed. Current studies show the effects of therapy on the contamination of the root canal biofilm with E. faecalis, demonstrating that photoactivated curcumin promotes the disruption of the biofilm and reduction of Colony-Forming Units.

**Conclusions:**

Curcumin as a photosensitizer demonstrates a potential antibacterial effect significantly decreasing the viability of microbial cells and the vitality of biofilms.

## INTRODUCCIÓN

En endodoncia, el avance de nuevos instrumentos y técnicas para el tratamiento del conducto radicular ha sido una característica predominante de la investigación y el desarrollo clínico durante los últimos 25 años ^1^. No obstante, si bien la terapia endodóntica exitosa puede definirse bien por la ausencia de periodontitis apical y síntomas clínicos después de un período de observación, el tratamiento fallido ha escapado a una posición distinta a lo largo de los años ^2^. 

En los últimos años, el desbridamiento químico y mecánico del conducto radicular son los métodos principales que se utilizan en la terapia endodóntica para eliminar todo el tejido muerto, las bacterias y los subproductos microbianos del conducto ^3^. Aunque el desbridamiento quimiomecánico tiene un papel esencial en el éxito del tratamiento de conductos radiculares, los estudios revelan que este método tiene ciertas limitaciones, ya que las biopelículas tienden a adherirse a las paredes del conducto radicular para formar centros bacterianos concentrados y pueden penetrar profundamente en las características anatómicas tales como los conductos accesorios, ramas apicales o túbulos dentinarios, especialmente en el tercio apical de los conductos radiculares ^4^. Por lo tanto, se requiere un nuevo enfoque no invasivo con alta eficacia y baja citotoxicidad para la eliminación de microorganismos patógenos del sistema de conductos radiculares ^5^.

El tratamiento fotodinámico se estableció en 1900 y ganó popularidad en la última década debido a sus beneficios para la erradicación de infecciones microbianas en endodoncia. Durante el tratamiento fotodinámico antimicrobiano, un fotosensibilizador no tóxico se activa mediante la irradiación de luz a una cierta longitud de onda en el tejido para producir especies reactivas de oxígeno y destruir los microorganismos ^6^. Este método de tratamiento se emplea en odontología para detener la propagación de los microorganismos que causan la periodontitis y las caries dentales. La no invasividad, la ausencia de necesidad de antibióticos y la posibilidad de erradicar rápidamente los microorganismos son solo algunas de las ventajas del tratamiento fotodinámico ^7^. 

La terapia antimicrobiana fotoactivada requiere tres componentes para iniciarse. El primero incluye el uso de un colorante como fotosensibilizante en el conducto radicular, que es activado por el segundo componente, que es una luz con longitud de onda dentro de la máxima absorción del fotosensibilizador, y el último es la presencia de oxígeno ^8^. Las especies de oxígeno reactivo, que pueden dañar los microorganismos y las células cancerosas, se producen cuando los fotosensibilizadores son activados por diferentes longitudes de onda de luz. La curcumina, la hipericina y la riboflavina son ejemplos de los nuevos fotosensibilizadores que entran en la categoría de sustancia natural ^9^. 

La curcumina posee una amplia gama de acciones biológicas, que incluyen características antivirales, antiinflamatorias, anticancerígenas y antibacterianas. El rizoma de la cúrcuma es de donde se obtiene principalmente la curcumina ^10^. Tiene propiedades antimicrobianas y, debido a su capacidad para absorber la luz azul y la producción de especies reactivas de oxígeno, se puede utilizar en la terapia fotodinámica. Un rango de longitudes de onda azules, principalmente entre 405 y 435 nm, es donde este fotosensibilizador resulta más activo ^9^.

La evidencia muestra que la curcumina puede inhibir la proliferación bacteriana cuando se irradia con una longitud de onda de luz específica ^11^. Además, la curcumina parece ser un candidato destacado que se puede utilizar en el tratamiento del osteosarcoma, al inhibir la progresión y reparar los defectos óseos simultáneamente ^12^.

Diversos estudios han determinado que la curcumina como fotosensibilizador activado por una longitud de onda azul tiene el potencial de eliminar diversas especies bacterianas involucradas en la enfermedad periodontal ^13^. Son pocos los estudios que evalúan el efecto antibacteriano de la terapia fotodinámica mediada por curcumina comparado con otros tratamientos complementarios. 

Por ello, el objetivo de esta revisión narrativa es actualizar e integrar la evidencia existente sobre la efectividad de la terapia fotodinámica utilizando curcumina para la reducción bacteriana en el tratamiento de conductos.

## MATERIALES Y MÉTODOS

### Estrategia de búsqueda

Se realizó un estudio de revisión bibliográfica que incluyó cinco bases de datos: PubMed/Medline, Scopus, EBSCO, ScienceDirect y LILACS como principales fuentes de información, publicados desde 2018 hasta marzo de 2023. La estrategia consideró descriptores Medical Subject Headings (MeSH) y palabras clave que fueron: (“curcumin” OR “curcuma”) AND (“photodynamic” OR “photochemotherapy” OR “phototherapy” OR “photoradiation” OR “light activated disinfection” OR “photoactivated disinfection” OR “laser-activated disinfection”) AND (“root canal disinfection” OR “root canal therapy” OR “root canal treatment” OR “endodontic treatment” OR “endodontic therapy” OR “root canal infection” OR “endodontic infection” OR “endodontics”). Además, las referencias fueron ordenadas mediante la búsqueda de citas RefWorks para evitar duplicados (Tabla 1).

**Tabla 1:** Estrategia de búsqueda de descriptores de las diferentes bases de datos

Medline/PubMed (22/03/2024)
n = 318
("curcumin” OR "curcuma") OR "photodynamic" OR "therapy" OR "photochemotherapy" OR "phototherapy" OR "photoradiation" OR "laser-activated disinfection" OR "photoactivated disinfection" OR " light activated disinfection") AND ("root canal therapy" OR "root canal treatment" OR "endodontic treatment" OR "endodontic therapy " OR "root canal infection" OR "endodontic infection" OR "endodontics" OR "root canal")
Scopus (22/03/2024)
n = 158
("curcumin” OR "curcuma") AND ("photodynamic" OR "therapy" OR "photochemotherapy" OR "phototherapy" OR "photoradiation" OR "disinfection" OR "photoactivated" OR " laser activated") AND ("root canal therapy" OR "root canal treatment" OR "endodontic treatment" OR "endodontic therapy " OR "root canal infection" OR "endodontic infection" OR "endodontics" OR "root canal")
EBSCO (22/03/2024)
n = 32
("photodynamic therapy" OR "phototherapy")AND("curcumin" OR "curcuma”) AND ("endodontic" OR "root canal")
ScienceDirect (22/03/2024)
n = 91
("photodynamic therapy" OR "phototherapy")AND("curcumin" OR "curcuma”) AND ("endodontic" OR "root canal")
LILACS (22/ 03/2024)
n = 150
("curcumin” OR "curcuma") AND ("photodynamic" OR "therapy" OR "phototherapy" OR "photoradiation" OR "laser activated" OR "disinfection" OR "photoactivated disinfection" OR "light activated disinfection") AND ("root canal therapy" OR "root canal treatment" OR "endodontic treatment" OR "endodontic therapy " OR "root canal infection" OR "endodontic infection" OR "endodontics") Filters: from 2019 - 2023

### Criterios de elegibilidad

Se incluyeron en la búsqueda estudios transversales o longitudinales, comparativos, ensayos clínicos, cohortes y casos y control, revisiones sistemáticas y metaanálisis pertenecientes desde 2016 a 2023, en idioma inglés y español. Los reportes de casos, editoriales, artículos de opinión, estudios en animales, estudios descriptivos, revisiones narrativas, cartas al editor, estudios duplicados y los que presentaban texto incompleto (“*no full text*”) estuvieron sujetos a criterios de exclusión. 

### Recolección de datos

**Figura 1:**
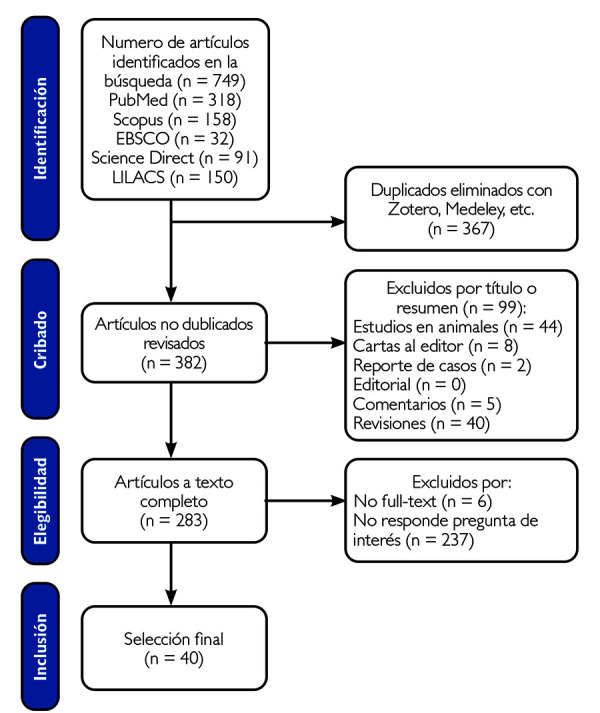
Diagrama de flujo PRISMA de la revisión de la literatura

Los estudios recolectados se analizaron los títulos y resúmenes según los criterios de elegibilidad. Esta revisión fue realizada por dos investigadores (A. S. V y M. Z. S) de forma independiente. De no haber resumen disponible, se evaluó el texto completo. Las dificultades relacionadas con la recopilación fueron resueltas por el segundo investigador (M. Z. S). Por último, se documentaron las causas de exclusión de los artículos que no cumplían con los criterios de selección establecidos. La elección de los artículos siguió el método PRISMA (Preferred Reporting Items for Systematic Reviews and Meta-Analyses) (Figura 1).

## RESULTADOS Y DISCUSIÓN

### Identificación de artículos

En la búsqueda se recolectaron 749 artículos distribuidos en PubMed (318), Scopus (158), EBSCO (32), ScienceDirect (91) y LILACS (150), y se encontraron 367 duplicados con Refworks. De los 382 artículos no duplicados, se excluyeron 99 por título o resumen (2 reportes de casos, 40 revisiones, 5 comentarios, 44 estudios en animales y 8 cartas al editor) y 283 en la revisión a texto completo, de los cuales 237 no respondieron la pregunta de interés y 6 fueron “*no full text*”. Finalmente, fueron seleccionados 40 artículos.

### Terapia fotodinámica con curcumina

La terapia fotodinámica antimicrobiana, también conocida como desinfección fotodinámica o quimioterapia antimicrobiana fotodinámica, implica la absorción de fotones de una cierta longitud de onda por un fotosensibilizador no tóxico con un espectro de absorción coincidente, lo que provoca la excitación de los electrones del estado fundamental ^16^^-^^18^. Uno de los fotosensibilizadores disponibles comercialmente es la curcumina, el cual es un compuesto natural con una banda de absorción entre 420 y 480 nm; por lo tanto, se activa con la luz azul ^19^. La curcumina contiene una variedad de acciones biológicas y farmacológicas, no es tóxica, es antiinflamatoria y antibacteriana. Además de esta amplia gama de efectos terapéuticos y sistémicos, la curcumina ha llamado recientemente la atención de varias organizaciones de terapia de fotones debido a su excelente actividad fotodinámica ^20^. En la Tabla 2 se presenta la recopilación de estudios previos en la efectividad de la terapia fotodinámica usando curcumina activada por luz y en la desinfección del conducto radicular con *E*. *faecalis*. Sin embargo, se ha demostrado, a partir de estudios recientes, sobre la desinfección de la dentina radicular con diferentes fotosensibilizadores son escasos ^(14, 15)^.

**Tabla 2:** Estudios que evaluaron la efectividad de la terapia fotodinámica usando usando curcumina en la desinfección del conducto radicular

Autor y año	Diseño del estudio	Tamaño de muestra	Grupos de Intervención	Láser	Tiempo de irradiación	Concentración	Resultado
Moradi et al. 2022	In vitro	63 dientes humanos	Grupo 1 - aPDT con curcumina Grupo 2 - aPDT con riboflavina Grupo 3 - LED Grupo 4 - Curcumina Grupo 5 - Riboflavina Grupo 6 - Grupo de control positivo (NaOCl al 5,25 %) Grupo 7 - Grupo de control negativo (sin tratamiento)	LED longitud de onda 390 - 480 nm	Irradiación de 5 minutos	10,2%	Con una reducción significativa del recuento de colonias de *E*. *faecalis*, aPDT con curcumina y riboflavina puede servir como complemento del método de desinfección de conducto radicular de rutina.
Oda et al. 2019	In vitro	80 incisivos bovinos	Grupo 1: control positivo Grupo 2: PDT estándar (azul de metileno + láser de diodo) Grupo 3: curcumina Grupo 4: luz de curado LED Grupo 5: curcumina + luz de curado LED	LED Longitud de onda 450 - 470 nm	Irradiación 5 minutos	20 umol/L	Se obtuvo una eficacia de desinfección similar usando curcumina más luz de curado LED y azul de metileno más LÁSER de 660 nm (PDT estándar).
Mozayeni et al. 2020	In vitro	54 dientes incisivos y premolares	Grupo 1: Irrigación (NaOCl) Grupo 2: irrigación (NaOCl+TB) Grupo 3: irrigación (NaOCl+MB) Grupo 4: irrigación (NaOCl+CUR) Grupo 5: Aplicación de disolvente curcumina (1% etanol+ disolvente CUR) durante 120 segundos	LED Longitud de onda 630 nm	Irradiación 2 minutos	0,5 mg/ml	La adición de TFD mediada por azul de toluidina con NaOCl aumentó su eficacia antibacteriana contra *E*. *faecalis* y podría ser un método complementario en la desinfección del conducto radicular.
Tellaroli et al. 2023	In vitro	72 muestras de dentina	Curcumina sin fotoactivación CUR+NP sin fotoactivación Curcumina fotoactivada CUR+NP sin fotoactivación	LED Longitud de onda 450 nm	Irradiación desde abajo 55 min	325 μg/mL de NP+CUR	La concentración de 325 μg/mL de NP+CUR fotoactivado fue la que más redujo la viabilidad de las bacterias endodónticas (*E*. *faecalis*, *S*. *oralis y A*. *viscosus*)

aPDT: Terapia fotodinámica antimicrobiana, CUR: curcumina, MB: azul de metileno, TB: azul de toluidina, TFD: terapia fotodinámica antimicrobiana, NP: nanopartícula polimérica, NaOCl: Hipoclorito sódico, nm: nanómetro, mg/ml: miligramos por mililitros, umol/L: micromol por litro, μg/mL: microgramo por mililitro.

### Acción antibacteriana

El uso de la curcumina en el tratamiento fotodinámico antimicrobiano se ha examinado durante los últimos 15 años y se ha encontrado que tiene un potencial significativo para la acción fotodinámica contra bacterias, hongos y cepas comunes resistentes a los medicamentos comunes ^21^. Además, existen estudios que evaluaron los efectos de la terapia fotodinámica antimicrobiana en la contaminación del biofilm del conducto radicular con *E*. *faecalis*, utilizando la curcumina como fotosensibilizante, llegando a la conclusión que la curcumina fotoactivada promovió tanto la ruptura completa de la estructura del biofilm como la reducción de unidades formadoras de colonias mL. Por ello, se informó que después del tratamiento fotodinámico con curcumina, se observó una alta tasa de destrucción del biofilm de *E*. *faecalis* y una disminución de la viabilidad en los conductos radiculares ^22^^-^^24^. En cambio, en otros estudios, los valores medios de unidades formadoras de colonias disminuyeron un 99% en comparación con el grupo de control ^25^.

### Tipo de irradiación

Por otro lado, un estudio que compara el efecto de la irradiación continua con baja intensidad y la irradiación fraccionada con alta intensidad reveló que la terapia fotodinámica mediada por curcumina en modo de irradiación continua promovió la reducción total de la carga microbiana; sin embargo, para el modo fraccionado, se requirió una mayor concentración de curcumina para reducir completamente la viabilidad celular de *E*. *faecalis*^26^. En otro estudio, en la irradiación de compuestos con 405 o 460 nm, solo la curcumina y el *Hypericum* alcanzaron la reducción logarítmica mínima generalmente reconocida para los procedimientos de asepsia ^27^. Estos compuestos presentaron un bajo riesgo de tinción dental, baja toxicidad oscura, buena producción de especies reactivas de oxígeno y un efecto antibacteriano adecuado, incluso en un desafío de biopelícula ^28^.

### Curcumina combinada

En un estudio realizado recientemente, se logró reducir la viabilidad bacteriana casi a la mitad en un tratamiento realizado durante 5 min con curcumina no fotoactivada sobre bloques de dentina infectados, en comparación con la del grupo control ^29^. Además, la combinación de curcumina con la luz LED azul reveló una inactivación bacteriana más eficaz, similar a la de la terapia fotodinámica antimicrobiana estándar realizada con láser de diodo y azul de metileno. Numerosas investigaciones han examinado los efectos de combinar la luz LED azul con diferentes tintes rojos y descubrieron que estas dos sustancias también pueden inactivar a las bacterias tanto en forma planctónica como de biopelícula ^30^. De igual forma, se demostró que la nanocurcumina y uno de los tintes, el verde de indocianina más metfromina, combinado con diferentes grupos de terapia fotodinámica antimicrobiana, logró una reducción significativa en las cepas de *E*. *faecalis* presentes en los conductos radiculares del 80% ^31^. Esto podría deberse a que la curcumina del estudio se activó usando un LED con una longitud de onda diferente (450 nm), lo que provocó que este fotosensibilizador absorbiera más luz ^32^.

La mezcla de curcumina con quelantes produce una disminución en la viabilidad y vitalidad de las capas internas de las biopelículas de E. *faecalis* mediante el empleo de terapia fotodinámica ^(33, 34)^. Debido a esto, las biopelículas en las cavidades óseas se pueden reducir de manera más efectiva usando este tratamiento con colorante de curcumina ^35^.

Sin embargo, en algunos estudios, el grupo tanino, mostró los mejores resultados como colorante sustituto con menor toxicidad y buena actividad antibacteriana comparada con el grupo curcumina que evidenció una marcada desviación estándar, y la reducción de bacterias del 95,4% no fue estadísticamente significativa ^36^. En contraste con los geles que contienen orégano, incienso y una combinación de aceites esenciales, el gel de curcumina tuvo una mayor viabilidad de los fibroblastos primarios de la pulpa dental y no mostró toxicidad en ninguna concentración. También tuvo el efecto inhibitorio más bajo. La falta de consideración por los espectros de absorción del gel de curcumina (437 nm) y la longitud de onda de la fuente de irradiación (LED 630 nm y láser 660 nm) condujeron a un impacto inhibidor disminuido ^37^.

### Nanopartículas de curcumina

Por otro lado, se ha evaluado la curcumina en forma de nanopartículas para superar sus limitaciones clínicas y disminuir su fototoxicidad. De esta manera, los hallazgos determinan que el uso de curcumina y nanocurcumina sola o en combinación con LED reduce drásticamente la concentración de *E*. *faecali* y mejora la limpieza del conducto radicular ^38^. Así mismo, en otro estudio se encontró que las nanopartículas poliméricas cargadas con curcumina tenían un efecto más fuerte que la curcumina sola en la erradicación de biopelículas de especies únicas y múltiples ^39^. También se descubrió que la luz LED azul mejoraba los efectos antibiofilm tanto de la curcumina como de las nanopartículas cargadas con curcumina ^40^.

Limitaciones: Como limitaciones del estudio declaramos que esta revisión no incluyó artículos en idiomas distintos al inglés o al español, por lo que es posible que hallazgos relevantes sobre el tema en otras lenguas no fueran considerados y, por lo tanto, se tiene una validez externa limitada. 

## CONCLUSIONES

En esta revisión, los estudios sugieren que la curcumina como fotosensibilizador demuestra un efecto antibacteriano potencial para disminuir significativamente la viabilidad de las células microbianas y la vitalidad de las biopelículas. Así mismo, se requieren más investigaciones a nivel clínico para poder determinar la eficacia antibacteriana de la curcumina en el tratamiento de conductos.
